# Generation Z’s attitude to urological work place environment—a pilot study

**DOI:** 10.3389/fmed.2026.1807563

**Published:** 2026-08-05

**Authors:** Yana Terziyska, Stefanie Cermak, Lujza Brunaiova, Lukas Koneval, Beat Roth, Laila Schneidewind

**Affiliations:** Department of Urology, University Hospital Bern, Inselspital, University of Bern, Bern, Switzerland

**Keywords:** gender, Generation Z, medical education, surgical education, urology

## Abstract

**Objective:**

It is known that Generation Z (GenZ) has different needs and expectations with regard to the world of work than previous generations. A secure working environment with long-term prospects must be created for them. These facts are essential for further improvement of urological education and residency. Thereof, we conducted a survey among Swiss urologists and urological residents.

**Design, setting and participants:**

Between July and September 2025 invitations to participate in our online survey were send. The pilot survey consisted of 93 items, e.g., the validated Workplace Gender Discrimination Scale.

**Results:**

Two-hundred-three questionnaires were received, leading to an overall response rate of 33.1%. Twenty (9.9%) of the respondents belonged to GenZ (all residents). Regarding the Workplace Gender Discrimination Scale GenZ had a median total score of 4.5. Only two persons (10.0%) from GenZ experienced a high level of discrimination. These facts were not significantly different from the previous generations (*p* = 0.418; *p* = 0.704), respectively. Regarding working conditions 13 (65.0%) of GenZ already experienced discrimination or disadvantage in their professional career, which is significantly more than in non-GenZ members (*p* = 0.009). However, we must assume that this study is not without limitations, like the response rate with only 20 GenZ members can lead to selection bias.

**Conclusion:**

Urological residents of GenZ have already experienced a significant higher level of discrimination than previous generations. This must be addressed in further research and development of structured residency programs to increase job satisfaction and therefor quality of patient care.

## Introduction

It is well known that Generation Z (born between 1996 and 2015) has different needs and expectations with regard to the world of work than previous generations (1, 2). They are willing to work with a high degree of ethics for the benefit of others, while at the same time being prepared to take on challenges. They have a strong sense of responsibility and commitment to long-term goals. Their desire for feedback and immediate response or proactive communication from supervisors is essential. Digital solutions for learning, work and communication are preferred (2–5). In summary, a secure working environment with long-term and sustainable prospects must be created for Generation Z (GenZ) and the integration of modern technologies into everyday working life is of enormous importance. The reasons for their priorities are not fully understood and further research is essential to understand their needs and professional characterization in detail. However, due to demographic change and the problem of recruiting young talents, we urologists are forced to improve medical and specialist training in order to ensure high-quality urological care based on the latest evidence and to increase the attractiveness of the profession (2). Unfortunately, the current evidence for GenZ working in urology is very limited, especially regarding workplace environment.

The workplace environment for both surgical and urological training encompasses the physical, organizational and social conditions that shape a trainee’s daily experience. This includes factors such as operating room atmosphere, workload, supervision quality, teamwork culture and broader institutional climate as well as ergonomic conditions and psychological safety. Together, these elements, ranging from the physical workspace to interpersonal relationships and organizational expectation, can strongly influence trainee wellbeing and learning outcomes. Ultimately, the workplace environment, including faculty culture, mentorship quality, team dynamics and institutional support, plays a central role in shaping learning outcomes as well as in patient outcomes or quality of care (6–13).

Consequently, we performed an analysis of our survey “Career pathways in urology in Switzerland—CaPu study” regarding workplace environment. The primary aim of this analysis was the detailed description of the impression of workplace environment for GenZ and thereof harvesting information to improve a safe educational atmosphere. Additionally, the secondary aim was the comparison to previous generations.

## Methods

### Development of the survey and target population

The pilot survey was designed by our working group after explorative literature search regarding workplace environment, clinical education and career pathways in PubMed via MEDLINE containing demographic characteristics of the target population, the validated Workplace Discrimination Scale and all aspects of workplace environment, notably working conditions, career opportunities, remuneration and recognition, working hours and work life balance, mentoring and leadership, experiences with discrimination and stereotypes as well as perception of equal rights in the facility.

It was designed and conducted according to the reporting guidelines for surveys found on the http://equator-network.org, an international initiative providing robust reporting and research guidelines (14, 15). The survey was only available in English and contained 93 items, 23 of these items were used for this specific analysis. Additionally, only complete questionnaires for these 23 items were used. Table 1 illustrates these items as well as the whole survey in detail.

**Table 1:** Survey items and items used for this analysis.

Category	Number of items in the initial survey “Career pathways in urology—CaPu”	Items used for this analysis
Demographic characterization	17	Gender Age Ethnicity Religion Sexual orientation Stable partnership Children in childcare University Diploma
Workplace Gender Discrimination Scale (validated)	5	5
Working conditions	9	Have there already been cases of discrimination or disadvantage in your professional career? (Yes/No) Have you already experienced sexual harassment in the course of your professional career, in particular by superior or colleagues? (Yes/No)
Career opportunities	8	Do you feel that your gender has influenced your career opportunities? (Yes/No)
Remuneration and recognition	4	Do you feel that there are differences in pay between male and female colleagues? (Yes/No)
Working hours and work life balance	19	Do you have difficulties balancing your working hours with family commitments? (Yes/No)
Mentoring and leadership	12	Do you have a mentor in your career? (Yes/No) Do you feel that women have the same access to mentoring opportunities as men? (Yes/No)
Experiences with discrimination and stereotypes	11	Have you ever felt that you are treated unfairly or stereotyped because of your gender? (Yes/No)
Perception of equal rights in the clinic/facility	7	Do you feel that you are treated equally when participating in important clinical or scientific projects? (Yes/No)
Additional open comments	1	Open comment regarding the survey

The Workplace Gender Discrimination Scale is a six-item scale that measures gender discrimination in the workplace through an evaluation of discrimination in the following areas: recruitment, promotions, pay, deployment, training and lay-offs (16–18). For our analysis we used only the first five items: men are recruited more easily than women, men are promoted more frequently than women, men are given more pay and benefits than women, men and women are allocated different jobs and men are given more opportunities for job development than women. Furthermore, the sum of all five questions were added for a total score between 0 (strongly disagree with all six items) and 18 (strongly agree with all six items). Then the participants were categorized into the following four groups: no gender discrimination in the workplace (when scoring 0 on the scale), low level of discrimination in the workplace (1–6), medium level of discrimination in the workplace (7–12) or high level of discrimination in the workplace (13–18).

The target population were urologists and residents in urology in Switzerland. After hand search of all relevant clinical societies and databases of urology in Switzerland 614 persons with mail addresses have been identified as survey population.

### Administration of the survey

The survey was transferred to the online platform SurveyMonkey® (Survey Monkey® by Momentive, CA, USA). It was tested for usability by the coauthors before setting it up online. The questionnaire was available from July 2025 to September 2025. An invitation or reminders were sent for four times to the mail addresses during that time frame. Additionally, the survey was advertised from the coauthors´ personal contacts and in the newsletter of the Swiss Society of Residents in urology (SRU).

The investigation was performed in accordance with the ethical standards of the institutional and national research committee and with the 1964 Helsinki Declaration and its later amendments or comparable ethical standards. Consent for this investigation for the human participants (also see target population) was not mandatory, and no personal data were stored throughout the process. Survey participation was voluntary, and no incentives were offered.

### Statistical analysis

For each numerical variable, the numerical distribution was preliminarily assessed by the Kolmogorov–Smirnov test. Descriptive statistics were made with means and standard deviations for normal distribution or with medians and IQRs for non-parametric data. For parametric continuous variables, Student’s *t* test was used, and for parametric categorical variables, the chi-square test or the Fisher exact test was used. For non-parametric data, the Mann–Whitney *U* test was used for categorical and continuous variables. All reported *p*-values were based on a two-sided hypothesis, and *p* < 0.05 was considered to be significant. All statistical calculations were performed using Statistical Package for the Social Sciences 29.0 software (SPSS Inc., IBM Corp., Armonk, NY, USA).

## Results

### Response rates and demographic characterization of the study population

Two-hundred-three questionnaires were received, leading to an overall response rate of 33.1%. Twenty (9.9%) of the respondents belonged to GenZ. The median age of GenZ was 29.0 years (IQR 28.0–30.0), 13 (65.0%) were male and 7 (35.0%) were females. There was no significant difference in terms of gender between GenZ and non-GenZ responders (non-GenZ male = 122; 66.7%; *p* = 0.881). All GenZ members were in urological residency. Only one member (5.0%) of GenZ had children in childcare. Table 2 illustrates the demographic characteristics of GenZ.

**Table 2:** Demographic characterization of GenZ (*n* = 20).

Item	*N* (%)	Median (IQR)
Gender	Male: 13 (65.0) Female: 7 (35.0)	–
Age	–	29.0 (28.0–30.0)
Ethnicity	European: 18 (90.0) Asian: 1 (5.0) Other: 1 (5.0)	–
Religion	Christian: 12 (60.0) Atheist: 7 (35.0) Muslim: 1 (5.0)	–
Sexual orientation	Heterosexual: 16 (80.0) Homosexual: 4 (15.0) Abstention: 1 (5.0)	–
Stable partnership	Yes: 18 (90.0) No: 2 (10.0)	–
Children in childcare	Yes: 1 (5.0) No: 19 (95.0)	–
University diploma	Switzerland: 10 (50.0) Germany: 3 (15.0) Austria: 2 (10.0) Other European country: 5 (25.0)	–

### Workplace Gender Discrimination Scale

**Figure 1:**
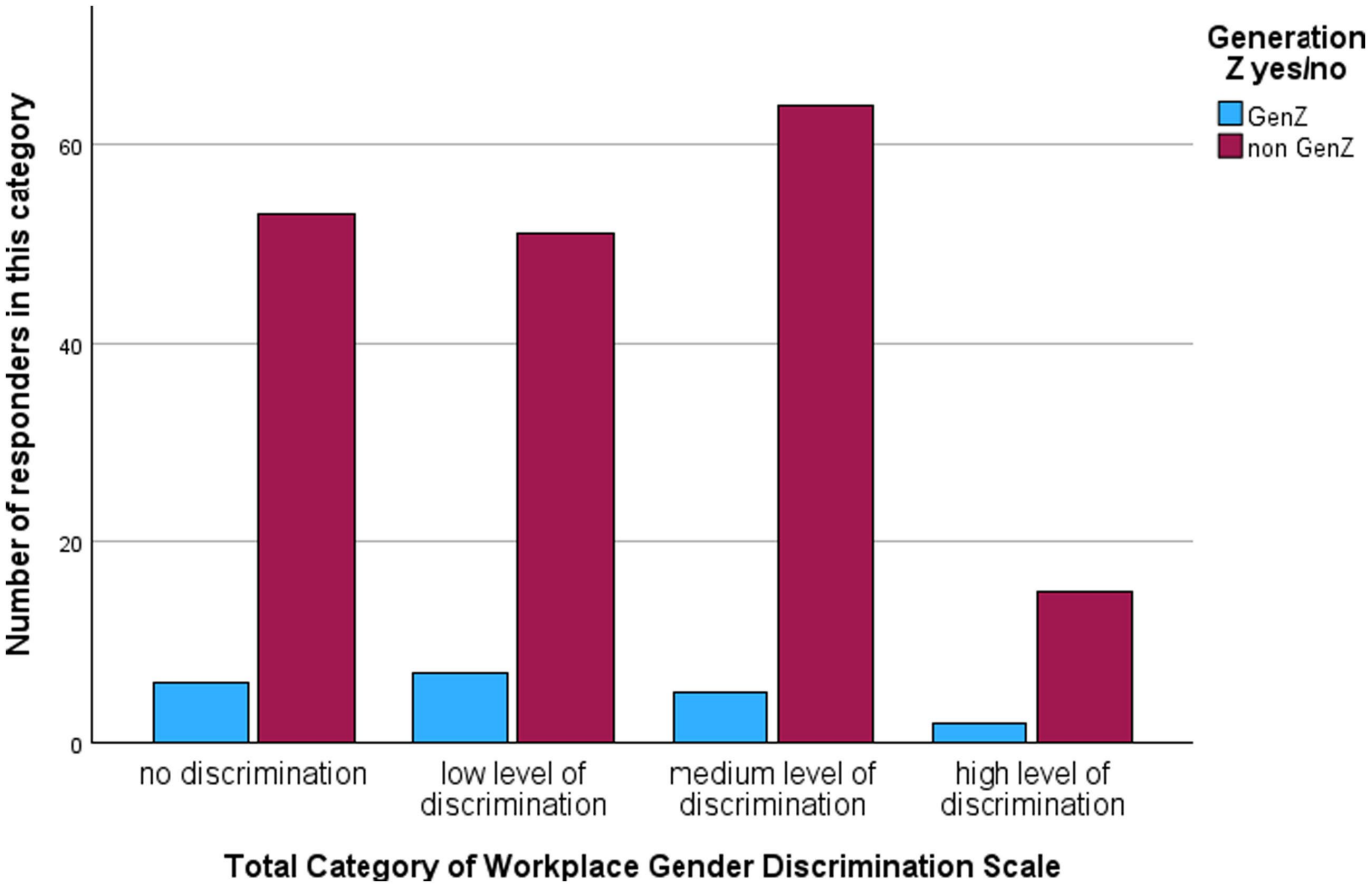
Total category of Workplace Gender Discrimination Scale, comparison between GenZ and non-GenZ.

Regarding the Workplace Gender Discrimination Scale GenZ had a median total score of 4.5 (IQR 0.0–7.0). Only two persons (10.0%) from GenZ experienced a high level of discrimination according to that score. These facts were not significantly different from the previous generations (*p* = 0.418; *p* = 0.704), respectively. Figure 1 illustrates the results of the Workplace Gender Discrimination Scale.

### Workplace environment

Regarding working conditions 13 (65.0%) of GenZ already experienced discrimination or disadvantage in their professional career, which is significantly more than in non-GenZ members (*p* = 0.009). In contrast, 16 (80.0%) of the GenZ members think that they are not treated equally when participating in important clinical or scientific projects. Interestingly, this is significantly different in the previous generations (*p* = 0.026). Table 3 shows the comparison between GenZ and non-GenZ of all aspects of workplace environment, notably working conditions, career opportunities, remuneration and recognition, working hours and work life balance, mentoring and leadership, experiences with discrimination and stereotypes as well as perception of equal rights in the department.

**Table 3:** Comparison regarding workplace environment between GenZ and non-GenZ members.

Category	Items	GenZ *N* (%)	Non-GenZN (%)	*p* value
Working conditions	Have there already been cases of discrimination or disadvantage in your professional career?	Yes: 13 (65.0) No: 7 (35.0)	Yes: 64 (35.0) No: 119 (65.0)	0.009
	Have you already experienced sexual harassment in the course of your professional career, in particular by superior or colleagues?	Yes: 2 (10.0) No: 18 (90.0)	Yes: 24 (13.1) No: 159 (86.9)	0.692
Career opportunities	Do you feel that your gender has influenced your career opportunities?	Yes: 2 (10.0) No: 18 (90.0)	Yes: 48 (26.2) No: 135 (73.8)	0.110
Remuneration and recognition	Do you feel that there are differences in pay between male and female colleagues?	Yes: 0 (0.0) No: 20 (100.0)	Yes: 28 (15.3) No: 155 (84.7)	0.060
Working hours and work life balance	Do you have difficulties balancing your working hours with family commitments?	Yes: 4 (20.0) No: 16 (80.0)	Yes: 68 (37.2) No: 115 (62.8)	0.128
Mentoring and leadership	Do you have a mentor in your career?	Yes: 4 (20.0) No: 16 (80.0)	Yes: 45 (24.6) No: 138 (75.4)	0.649
	Do you feel that women have the same access to mentoring opportunities as men?	Yes: 5 (25.0) No: 15 (75.0)	Yes: 73 (39.9) No: 110 (61.1)	0.194
Experiences with discrimination and stereotypes	Have you ever felt that you are treated unfairly or stereotyped because of your gender?	Yes: 1 (5.0) No: 19 (95.0)	Yes: 26 (14.2) No: 157 (85.8)	0.250
Perception of equal rights in the clinic/facility	Do you feel that you are treated equally when participating in important clinical or scientific projects?	Yes: 4 (20.0) No: 16 (80.0)	Yes: 84 (45.9) No: 99 (54.1)	0.026

### Additional comments

One comment from GenZ was that the initial survey was way to long and not very well structured. This was also stated by 10 non-GenZ members. However, one member of the previous generation commented that in the last 30 years there were a lot of changes regarding workplace environment and education. Interestingly, one non-GenZ participant stated that this is a very important topic, especially for residency, while one non-GenZ member said that the survey had nothing to do with education or residency at all. Additionally, two non-GenZ participants stated that childcare is the most important topic for women in this context. Lastly, one non-GenZ member thinks that the reduction of working hours in Switzerland would be the best start to improve workplace environment.

## Discussion

To the best of our knowledge, we conducted the first detailed analysis of GenZ’s attitude to urological workplace in Switzerland. The results are of particular importance since GenZ is more relying on a secure working environment with long-term and sustainable prospects than previous generations (2). Additionally, the workplace environment, e.g., mentorship quality, plays a central role in shaping learning outcomes as well as in patient outcomes (6–13). Due to demographic development and shortage of young talents even urologists must face these facts. However, the advantage of the gained knowledge from this analysis is the information to improve working environment for GenZ and therefore, ensuring quality of patient care.

Regarding, the Workplace Gender Discrimination Scale 10.0% of GenZ experienced a high-level of gender discrimination. Furthermore, gender discrimination is not significantly different to previous generations according to that score. Still, this number sounds low, but exposure to any form of violence, e.g., any kind of discrimination or sexual harassment, at work can lead to burnout and posttraumatic stress disorders (6). Sexual harassment also impacts teamwork as well as communication and so potentially compromises patient care (13). Consequently, heads of residency programs must be aware of harassment for all genders and must have plans to tackle this to ensure a save working atmosphere. Interestingly, literature data also suggests that career advancement is associated with less exposure to violence (6). Johnson et al. (9) also reported higher levels of burnout in younger surgeons than in older ones. Therefore, it could be assumed that GenZ is most vulnerable in this context and might need special protection. Another relevant aspect in this context is the limited availability of female mentors and role models for women in urology, particularly for residents and early-career urologists (6, 8, 11). However, the issue extends beyond gender-concordant mentoring. Our data indicate a broader structural deficit in mentorship quality and availability: 80.0% of GenZ respondents reported lacking an adequate mentor to support their career development. This underscores the need for committed, high-quality mentors across all genders to ensure effective professional guidance and longitudinal career support.

Unfortunately, 65.0% of GenZ have already experienced discrimination or disadvantage in their professional career, which is significantly higher than in previous generations (*p* = 0.009). On the one hand, the explanation is not quite obvious and it might be due to the fact that GenZ has a different perception about the workplace environment than previous generations. This must be addressed in further research, especially in urological care. On the other hand, a safe educational environment is essential and so this must be taken serious in developing structured residency programs in urology. Brown et al. (7) stated it this way: A training environment that focusses on role-modelling and experimental learning with adequate time for reflection and debriefing is paramount. In our opinion, this reflection also includes personal interaction, communication and fairness. Furthermore, 80.0% of GenZ reported that they are not treated equally in participating in important clinical or scientific projects, which is also significantly different than in previous generations (*p* = 0.026). Again, this might be due to different perception in the working context by GenZ members, but still they state that they are not treated fairly during their residency, which should be taken seriously by educators and mentors. In our mind, this should also be addressed in routine residency evaluations since they serve structure and process quality (10). Siech et al. underlines also the importance of structured education for residents and setting as well as revisiting specific goals. This can be essential for achieving academic and career objectives and thereby, increasing job satisfaction. Mandatory interim discussion at pre-defined dates could be a key to enhancing job satisfaction and to optimizing learning success (10). Logically, this might also lead to optimized patient care.

Interestingly, the other aspects of workplace environment, like mentoring and leadership or remuneration and recognition are not significantly different between GenZ and previous generations. Still, GenZ experience discrimination and this needs further evaluation, especially because this generation is relying on a safe environment. Additionally, this aspect must be integrated in the development of new structured urological residency programs for this generation.

Regarding the additional comments, one aspect must be discussed in detail: the allocation of time for childcare for all genders. In this survey only one GenZ member had children in childcare, but as time approaches it become an issue also for this generation. Wiemer et al. concluded from their detailed survey of female urologists in Germany: The fact that starting a family for women in urology in Germany is associated with a reduction in working hours and the end of clinical careers suggests a need for optimizing the compatibility of family and career in Germany. The increasing feminization of the medical profession exacerbates the existing shortage of skilled workers due to the exit of mothers from professional life. To meet the needs of working parents, particularly mothers, urgent adjustments in the work environment are necessary. Promoting flexible working time models and creating supportive conditions are crucial to preventing the loss of skilled professionals and maintaining job satisfaction in this field (8). Naturally, this should apply to all genders having children in childcare. Due to demographic development urological professionals are desperately need, consequently flexible working models are needed to ensure a high job satisfaction and keep these professionals employed.

However, we must assume that this study is not without limitations, like the very response rate with only 20 GenZ members can lead to selection bias. Furthermore, the results only apply for a European countries like Switzerland and cultural differences must also taken into account, concerning workplace environment. Then we must agree with the criticism, that we first conducted a very long and complicated survey to harvest a lot of preliminary data, but further research must be more precise and focused. Then, we did not consider psychological aspects like perception of communication for this survey. The topic of GenZ residency is highly emotionally charged and characterized by markedly divergent opinions, a finding that is also clearly reflected in the free-text comments provided by respondents. Establishing a solid scientific evidence base as well as theoretical psychological framework are therefore essential to move beyond anecdotal impressions and polarized viewpoints and to enable an objective, data-driven assessment. Unfortunately, data in the work field of medicine, especially urology, are really sparse. Additionally, we did not use the last item of the Workplace Gender Discrimination Scale, mainly due to the fact of the extent of our pilot survey. Lastly, we did not perform subgroup analysis regarding gender aspects, especially due to low number of GenZ members, but this must be addressed in further research since the gender might have significant influence on workplace environment perception and educational aspects. Additionally, this research must be extended to a non-binary gender definition.

In contrast, this study provides first information about the attitude to workplace environment of GenZ in urology, which can help to develop tailored residency programs ensuring also job satisfaction for this generation and therefore help to improve patient care in a time of higher working requirements for health care professionals.

## Conclusion

GenZ has different needs regarding workplace environment than previous generations, especially a secure working environment with long-term and sustainable prospects must be created for them. Urological residents of GenZ have already experienced a significant higher level of discrimination than previous generations. This must be addressed in further research and development of structured residency programs to increase job satisfaction and therefore quality of patient care.

## Data Availability

The original contributions presented in the study are included in the article/supplementary material, further inquiries can be directed to the corresponding author.
